# Depression and its associated factors among people living with HIV in the Volta region of Ghana

**DOI:** 10.1371/journal.pmen.0000035

**Published:** 2024-06-04

**Authors:** Jerry John Nutor, Robert Kaba Alhassan, Rachel G. A. Thompson, David Ayangba Asakitogum, Henry Ofori Duah, Tiarney D. Ritchwood, Nkothula Nkosi, Ntombifikile Klaas, Sampson Opoku Agyemang, Akua O. Gyamerah

**Affiliations:** 1 Department of Family Health Care Nursing, School of Nursing, University of California, San Francisco, San Francisco, California, United States of America; 2 Centre for Health Policy and Implementation Research, Institute of Health Research, University of Health and Allied Sciences, Ho, Ghana; 3 Language Center, College of Humanities, University of Ghana, Accra, Ghana; 4 Africa Interdisciplinary Research Institute, Accra, Ghana; 5 College of Nursing, University of Cincinnati, Cincinnati, Ohio, United States of America; 6 Department of Social Sciences and Health Policy, Wake Forest University, Winston-Salem, North Carolina, United States of America; 7 Department of Nursing Education, University of the Witwatersrand, Johannesburg, South Africa; 8 School of Nursing and Midwifery, University of Cape Coast, Cape Coast, Ghana; 9 Department of Community Health and Health Behavior, University of Buffalo, Buffalo, New York, United States of America; The University of Waikato, NEW ZEALAND

## Abstract

Depression among people living with HIV/AIDS in higher-income countries is associated with suboptimal adherence to antiretroviral therapy and though counterintuitive. Yet, less is known regarding how depression, social support, and other sociodemographic factors influence outcomes among people living with HIV, particularly in resource-limited settings like Ghana. In view of this gap, this study investigated factors associated with depressive symptoms among people living with HIV in the Volta region of Ghana. A total of 181 people living with HIV from a local antiretroviral clinic was purposively sampled for the study. The questionnaire included the Center for Epidemiologic Studies Depression Scale, the Internalized Stigma of HIV/AIDS Tool, and the Interpersonal Support Evaluation List-12. An independent student t-test, one-way analysis of variance, and chi-square test were conducted to ascertain the associations among the variables of interest. The magnitude of association was evaluated with multiple linear regression. The average depression score among the participants was 9.1±8.8 and 20.4% reported signs of depression. Majority (78%) of participants who were depressed were male compared to females (p = 0.031). In the multiple linear regression, every one-year increase in age was significantly associated with an estimated 0.012 standard deviation increase in depression scores (95% CI: 0.002–0.021) after adjusting for all other variables in the model. Every unit standard deviation increase in social support was significantly associated with an estimated 0.659 standard deviation increase in depression scores (95% CI:0.187–1.132), after adjusting for all other variables in the model. We found a high prevalence of depressive symptoms among people living with HIV especially among males. An increase in age and social support was associated with an increase in depressive symptoms among people living with HIV in this study. We recommend further study using longitudinal approach to understand this unexpected association between depression and social support among people living with HIV in Ghana.

## Introduction

Globally, people living with HIV (PLWH) face numerous barriers to engagement and retention in HIV care, including inconsistent access to antiretroviral therapy (ART) access and suboptimal healthcare utilization [1]. Common barriers to ART adherence in both low-income and high-income countries, for example, include depression, substance abuse, stigma, social support, and the side effects of ART drugs [1–3]. In addition, studies conducted in sub-Saharan Africa reported financial problems, drug toxicity, and forgetting to take medication as reasons for poor retention in HIV care [4, 5]. Interventions such as free ART services, family support, collaborative provider-patient relationships, and peer support were found to be effective measures in mitigating the barriers to ART adherence [3, 6–8]. However, these interventions did not improve ART adherence among PLWH who were depressed [9].

Depression is described as extreme sadness with symptoms such as anhedonia, hopelessness, poor concentration, and fatigue [10, 11]. These symptoms can potentially diminish one’s self-efficacy and motivation to engage in healthy behaviors including seeking health care and adhering to prescribed treatment regimens [11]. In South Africa, depression is found to be highly prevalent among PLWH and is associated with suboptimal ART adherence [7]. PLWH who reported anxiety and depressive symptoms were identified to be non-adherent to their treatment regimens than those without anxiety and depressive [10, 12]. Similarly, a study in South Africa found that depression prevalence was higher with poorer adherence to ART among PLWH who received case management than those who did not receive case management [13]. Studies have attributed the causes of depression among PLWH to their uncertainty of the future, physical and financial problems, social isolation, and stigma [14–16].

In sub-Saharan Africa, studies have reported a range of high prevalence rates of 24.5% [17] to 56.7% [18] of depression among PLWH. Factors associated with depression in PLWH in sub-Sharan Africa are inconsistent. While some studies reported poor treatment adherence [19] and being female [17, 18] as risk factors associated with depression, one study reported good treatment adherence and being a male [20] as factors associated with depression. Other risk factors associated with depression in PLWH included substance use [17, 19, 21], having low socioeconomic status [17, 18, 21], and being divorced and older than 60 years [17]. While the prevalence rate of depression in PLWH is on the rise [18] with inconsistent risk factors, the factors associated with depression in this population have not received attention in Ghana. In Ghana, the prevalence of depression in PLWH can be as high as 53.4% [22]. In addition, PLWH who have depressive symptoms in Ghana are at risk of neurocognitive impairment [23]. Two qualitative studies identified ART adherence facilitators in Ghana to include having access to food, disclosure of HIV status, availability of ART, family and partner support, organization of ART clinics, and transportation cost [24, 25]. However, the literature suggests that the facilitators of ART adherence may not benefit PLWH who have depressive symptoms.

Therefore, there is a paucity of empirical data on the factors associated with depression among PLWH in Ghana. However, the evidence of depression among PLWH could hinder ART adherence. A better understanding of the associated factors of depression is critical to guide effective ART treatment implementation and provide supportive care for PLWH. Our study, thus, investigated the factors associated with depression among PLWH in the Volta region of Ghana.

## Methods

### Study setting and design

This was a hospital-based analytic cross-sectional survey of PLWH who were enrolled in ART. The study was conducted in t a major referral hospital in the Volta Region of Ghana. Ghana has an HIV prevalence rate of 1.7% and the Volta Region is one of the regions with a high HIV prevalence rate of 1.3% [26]. The hospital has an ART clinic that serves as the referral center for HIV treatments and care services in the Volta region of Ghana. The ART clinic has a patient population of over 1000 PLWH. Using purposive sampling, we recruited and collected data from 181 PLWH who received care at the ART clinic between November 2021 and March 2022. See S1 Checklist for inclusivity in global research questionnaire.

### Recruitment and data collection

PLWH who received care at the selected clinic were contacted in person by a nurse when they arrived for a clinic visit to obtain their antiretroviral medication or for their regular appointment with their healthcare providers. The attending nurse briefly discussed the study with potential participants. Those PLWH who agreed to learn more about the study were contacted by a trained research assistant who is fluent in both English and the local language (Ewe). The research assistant explained the purpose, benefits, risks, and confidentiality to potential participants in either English or Ewe based on the participant’s preference. A copy of the study’s information sheet was given to the potential participants who could read it. For the PLWH who could not read, the research assistant explained the information sheet in a local language that they could understand. Potential participants were told that participation in the study is voluntary and that refusal to participate will not affect their access to care. Those who agreed to participate in the study were given a written informed consent form to sign. The eligible criteria included PLWH who were age 18 years or older, tested positive for HIV for at least 6 months, and consent to participate in the study. We excluded PLWH who were seriously ill, defined as hospitalization at the time of the data collection, and those who were diagnosed with mental illness. These eligibility criteria helped to include potential participants who have been living with HIV for a longer period and able to participate in the study. All eligible potential participants approached agreed and consented to participate in the study. Participants received the Ghana Cedis equivalence of $10 compensation after the survey administration for their time and travel costs. Data was collected between November 2021 and March 2022

### Sample size determination

Given our interest in understanding the impact of depression on social support among PLWH, we assumed α = 0.025, a Bonferroni correction for two tests. Using Stata/IC v16.1 [27] and assuming a depression prevalence of 30%, with 160 people, the minimum detectable odds ratio is 3.1, equivalent to an approximately medium effect size [28].

### Dependent variable

#### Depression

Risk of depression was measured with the Center for Epidemiologic Studies Depression Scale (CES-D) [29]. The CES-D is a 20-item Likert tool that evaluates the number of days in the past week during which individuals felt depressed. The response options for each item range from 0 (rarely or none of the time) to 3 (most or all the time). The CES-D scores range between 0 and 60, with high scores indicative of a greater risk for depressive symptoms. Depression scores were categorized using the cutoff point of 16 [30]. Participants with CES-D scores less than 16 were defined as having a low risk of depressive symptoms and those with CES-D scores ≥ 16 were described as having a high risk of depressive symptoms. Total depression scores were standardized before performing multiple regression analyses. Inter-item reliability analysis for our sample data revealed an acceptable Cronbach’s alpha (0.87).

### Independent variables

#### Social support

Social support was measured with the Interpersonal Support Evaluation List-12 (ISEL-12) [31]. The ISEL-12 tool comprises 12 items rated on a 4-point Likert scale that assesses perceived social support by asking respondents about their ability to find assistance in various social conditions of need. The scores of the responses range from 1(definitely false) to 4 (definitely true*)*. Six items were reverse scored (Items 1, 2, 7, 8, 11, and 12). High scores indicate a high level of perceived social support. The total scores of ISEL-12 tool range from 12–48. Moreover, the ISEL-12 tool can be divided into three subdomains, each consisting of 4-items: Appraisal Support, Belonging Support and Tangible Support. The scores of all the three subdomains ranges 4–16. The median of the total ISEl-12 score of this sample was 17. Scores lower than 17 were categorized as weak/low social support and scores of 17 and above were categorized as high social support. Inter-item reliability test of our sample data was performed, which revealed a Cronbach’s alpha value of 0.82. Scores were standardized prior to inclusion in multiple regression analysis.

#### Stigma

Stigma was measured with the Internalized Stigma of HIV/AIDS Tool (ISAT) [32]. The ISAT scale is a 10-item tool that evaluates the negative self-perceptions of individuals in relation to HIV. The 10 items on the ISAT are evaluated on a five-point Likert scale ranging from 1 (strongly disagree) to 5 (strongly agree). High total ISAT scores are indicative of a high level of internalized stigma. In the bivariate analysis, the ISAT scores were categorized based on the median score of the sample (median = 27), with low stigma being ISAT scores below 27 and high stigma defined as ISAT scores greater than or equal to 27. The ISAT scores were standardized prior to multiple regression analysis. We performed an inter-item reliability test in our sample and found that the ISAT had a Cronbach’s alpha value of 0.85.

#### HIV status disclosure

Participants were asked whether they had disclosed their HIV status to their current or past sexual partner.

#### Covariates

In our multivariable analysis, we adjusted for the following covariates: relationship status, rural/urban residence, gender, age, level of education, monthly income, and disclosure of HIV status to partner. Stigma was measured with the Internalized Stigma of HIV/AIDS Tool (ISAT) (Table 1).

**Table 1:** Sample characteristics (N = 181).

Variables	n	%
**Relationship Status**		
Single/Widowed	79	43.6
In Relationship	95	52.5
*Missing*	7	3.9
**Residence**		
Rural	86	47.5
Urban	90	49.7
*Missing*	5	2.8
**Gender**		
Female	25	13.8
Male	101	55.8
*Missing*	55	30.4
**Age**		
>30 years	15	8.3
30–39	35	19.3
40–49	55	30.4
50–59	45	24.9
60+	25	13.8
*Missing*	6	3.3
**Level of Education**		
No Formal education	18	9.9
Primary	86	47.5
Secondary	49	27.1
Post-Secondary	21	11.6
*Missing*	7	3.9
**Monthly Income**		
<1000GH	135	74.6
>1000 GH	25	13.8
*Missing*	21	11.6
**Disclosure of HIV Status**		
Disclosed	84	46.4
Have not disclosed	85	47.0
*Missing*	12	6.6
**Total Depression Score** (n = 170)	9.11±8.79[0–35]	
**Depression Score**		
0–15	133	73.5
16 and above	37	20.4
*Missing*	11	6.1
**Stigma Score**		
low stigma	82	45.3
Score 27 and above: high stigma	88	48.6
*Missing*	11	6.1
**ISEL-12**		
Low Social Support	83	45.9
High Social Support	87	48.1
*Missing*	11	6.1
Appraisal Support (n = 172)	5.96±2.57[4–13]	
Belonging Support (n = 172)	6.68±2.87[4–16]	
Tangible Support (n = 170)	6.08±2.39[4–14]	
ISEL 12 Total (n = 170)	18.67±6.61[12–41]	

Categorical variables are reported as frequency (%) and continuous variables are reported as mean± SD [Min-Max]

#### Ethics statement

Ethical approval for the study was obtained from the University of Health and Allied Sciences (UHAS) Research Ethical Committee with reference number UHAS-RECA.6 [1] 20–21 and the University of California San Francisco Institutional Review Board with reference number 20–32955. Permission was also sought from the management of the Ho Teaching Hospital and the HIV Clinic. Confidentiality was ensured at all stages of the process. Respondents gave written informed consent prior to enrollment using the approved consent forms. Respondents were assured that refusal to participate or withdrawal from the study would not affect their access to healthcare services at the clinic.

### Data analysis

We performed univariate, bivariate, and multivariable analyses. Frequency and percentages were reported for categorical variables whereas averages and standard deviations were reported for continuous variables in the univariate and bivariate analysis. Test of association was performed using independent samples t-test, ANOVA, and chi-square test for independence. The magnitude of association was evaluated with multiple linear regression. Missing data categories were included in the univariate and bivariate analyses. Standardized values of social support, stigma, and depression scores were used in multiple regression modeling. The analytical sample for the univariate, bivariate, and multivariable analysis were 181,170 and 111, respectively. Statistical significance was set at an alpha level of 0.05. We reported regression coefficients, confidence intervals, and p-values. All analysis was performed with Stata 14 software (StataCorp, 2015).

## Results

### Sociodemographic characteristics

A total of 181 participants were included in this study with a majority (≈ 60%) being male. Approximately half of the participants resided in urban areas. About 53% of the participants were in a relationship at the time of the survey with 44% being single/widowed. Cumulatively, 55% of participants were in the 40–59 years with 13.8% aged 60 years and above. Approximately 48% of participants had primary education, 27.1% had high school education, and 11.6% had attained a college education or higher. A majority (≈75%) earned less than GH1000 (USD 100) equivalence for the sake of international readers) monthly income and 47% indicated they had not disclosed their HIV status to their partners.

### Prevalence estimates of stigma, depression, and social support

Approximately 49% [95% CI:41.3%, 55.9%] reported high stigma with an average stigma score of 26.4 ± 8.5. The average depression score was 9.1±8.8 and 20.4% [95%CI:14.5%, 26.3%] reported signs of depression. The average total social support score was 18.7±6.6 with 45.9% [95% CI:38.6%, 53.2%] reporting low social support (Table 1).

### Depression and related factors

In bivariate analysis, we found that a majority (78%) of participants who were depressed were males compared to 2% of females (p = 0.031). Among those who were depressed 83.8% earned less than GH1000 monthly revenue whereas 75.6% of the non-depressed participants earned less than GH1000 (p = 0.036). Approximately 92% of participants who reported depressive symptoms had high social support compared to 40% of those without symptoms of depression (p<0.001). Likewise, 100% of the participants who were depressed had high stigma compared to 38.3% of those with no depressive symptoms (p<0.001), Table 2.

**Table 2:** Sample characteristics by depression status (N = 170).

	Not depressed	Depressed	Total	
	(N = 133)	(N = 37)	(N = 170)	p-value
**Relationship Status**				0.611
Single/Widowed	58 (43.6%)	18 (48.6%)	79 (43.6%)	
In Relationship	74 (55.6%)	19 (51.4%)	95 (52.5%)	
*Missing*	1 (0.8%)	0 (0.0%)	7 (3.9%)	
**Residence**				0.790
Rural	65 (48.9%)	19 (51.4%)	86 (47.5%)	
Urban	68 (51.1%)	18 (48.6%)	90 (49.7%)	
*Missing*	0 (0.0%)	0 (0.0%)	5 (2.8%)	
**Disclosure of HIV Status**				0.912
Disclosed	65 (48.9%)	17 (45.9%)	84 (46.4%)	
Have not disclosed	66 (49.6%)	18 (48.6%)	85 (47.0%)	
*Missing*	2 (1.5%)	2 (5.4%)	12 (6.6%)	
**Gender**				0.031
Female	23 (17.3%)	2 (5.4%)	25 (13.8%)	
Male	72 (54.1%)	29 (78.4%)	101 (55.8%)	
*Missing*	38 (28.6%)	6 (16.2%)	55 (30.4%)	
**Age**				0.576
Under 30	12 (9.0%)	3 (8.1%)	15 (8.3%)	
30–39	26 (19.5%)	8 (21.6%)	35 (19.3%)	
40–49	44 (33.1%)	8 (21.6%)	55 (30.4%)	
50–59	34 (25.6%)	10 (27.0%)	45 (24.9%)	
60+	17 (12.8%)	8 (21.6%)	25 (13.8%)	
*Missing*	0 (0.0%)	0 (0.0%)	6 (3.3%)	
**Monthly Income**				0.036
<1000 Cedis	101 (75.9%)	31 (83.8%)	135 (74.6%)	
>1000 Cedis	22 (16.5%)	1 (2.7%)	25 (13.8%)	
*Missing*	10 (7.5%)	5 (13.5%)	21 (11.6%)	
**Social Support Mean (SD)**	16.9 (5.7)	25.1 (5.7)	18.7 (6.6)	<0.001
**Social Support level**				<0.001
Low	80 (60.2%)	3 (8.1%)	83 (45.9%)	
High	53 (39.8%)	34 (91.9%)	87 (48.1%)	
*Missing*	0 (0.0%)	0 (0.0%)	11 (6.1%)	
**Stigma**				<0.001
Low	82 (61.7%)	0 (0.0%)	82 (45.3%)	
High	51 (38.3%)	37 (100.0%)	88 (48.6%)	
*Missing*	0 (0.0%)	0 (0.0%)	11 (6.1%)	

Data source: Field data (November 2021 and March 2022)

### Independent predictors of depression

Compared to those who have disclosed their HIV status, non-disclosure of status was estimated to be associated with a 0.301 standard deviation decrease in depression scores (95% CI: 0.551–0.052) after adjusting for all other variables in the model. Relative to females, being a male was associated with an estimated 0.405 standard deviation increase in depression scores (95% CI: 0.079–0.732), after adjusting for all other variables in the model.

Compared to those with no formal education, secondary education was associated with an estimated 0.442 standard deviation increase in depression scores (95% CI: 0.04 to 0.844), after adjusting for all other variables in the model. Every one-year increase in age was associated with an estimated 0.012 standard deviation increase in depression scores (95% CI: 0.002–0.021) after adjusting for all other variables in the model. Every unit standard deviation increase in stigma was associated with an estimated 0.405 standard deviation increase in depression scores (95% CI:0.236–0.577), after adjusting for all other variables in the model. Every unit standard deviation increase in social support was associated with an estimated 0.659 standard deviation increase in depression scores (95% CI:0.187–1.132), after adjusting for all other variables in the model (Table 3)

**Table 3:** Multiple regression estimates of depression.

Depression Score	Coef.		St.Err.	t-value		p-value	[95% CI Lower	Upper	Sig
**Relationship Status**									
Single/Widowed	*ref*		-	-		-	-	-	
In Relationship	0.111		0.134	0.83		0.41	-0.155	0.376	
**Residence**									
Rural	*ref*		-	-		-	-	-	
urban	-0.004		0.109	-0.04		0.969	-0.22	0.211	
**Disclosure of HIV Status to Partner**									
Disclosed	0		-	-		-	-	-	
Have not disclosed	-0.301		0.126	-2.40		0.018	-0.551	-0.052	**
**Gender**									
Female	*ref*		-	-		-	-	-	
Male	0.405		0.164	2.47		0.015	0.079	0.732	**
**Level of Education**									
No formal Education	*ref*		-	-		-	-	-	
Primary	0.018		0.181	0.10		0.921	-0.342	0.377	
Secondary	0.442		0.203	2.18		0.032	0.04	0.844	**
Post-Secondary	0.187		0.249	0.75		0.453	-0.306	0.681	
**Age**	0.012		0.005	2.47		0.015	0.002	0.021	**
**Monthly Income**									
<1000 Cedis	*ref*		-	-		-	-	-	
>1000 Cedis	0.214		0.185	1.16		0.25	-0.154	0.583	
**Total Stigma**	0.407		0.086	4.73		<0.001	0.236	0.577	***
**Total Social Support**	0.659		0.238	2.77		0.007	0.187	1.132	***
**Appraisal Support**	-0.209		0.144	-1.45		0.149	-0.494	0.076	
**Belonging Support**	0.141		0.151	0.93		0.353	-0.159	0.44	
Constant	-0.92		0.39	-2.36		0.02	-1.695	-0.146	**
Mean dependent var		-0.040			SD dependent var			1.048	
R-squared		0.755			Number of obs			110	
F-test		22.750			Prob > F			0.000	
Akaike crit. (AIC)		194.779			Bayesian crit. (BIC)			232.585	

*** p < .01

** p < .05

* p < .1

## Discussion

Our study examined the associated factors of depressive symptoms among PLWH in the Volta region of Ghana. Overall, we found that the prevalence estimates of depressive symptoms among PLWH was 20.4%. We also found that depressive symptoms were higher among men compared to women. PLWH who did not disclose their HIV status to family members or friends were less likely to report depressive symptoms compared to those who disclosed their status.

The prevalence of depression is lower in our study compared to findings reported in a study conducted in the central region of Ghana in which the prevalence of depression was about 50% and about 72% of PLWH had other mental health disorders [33]. However, higher than the rate (18.7%) reported in another study among PLWH receiving ART at the Korle Bu Teaching Hospital in Accra, Ghana [34]. Plausibly, the difference is attributable to variations in study design and depression measures. However, the findings corroborate the results of other hospital based studies in Eastern Africa in which 20% depression prevalence was reported among PLWH [35].

We found that being a male was positively associated with depressive symptoms in PLWH. This finding contradicts a previous study among PLWH in Ghana which reported higher levels of depression in females than their male counterparts [34]. Moreover, this finding is consistent with a study in Vietnam that found that among PLWH, men report less depressive symptoms compared to women [16]. In another study in sub-Sharan Africa, researchers found no significant association between gender and depression [36, 37] Thus, there is a need for further research to explore the prevalence and differential impact of depression by gender among PLWH.

Regarding HIV status disclosure, we found that non-disclosure was negatively associated with depressive symptoms which suggests that non-disclosure was protective against depression. Disclosure of HIV status is usually associated with unwarranted discrimination, shame, hostility, marital issues, and other social stigma in Ghanaian communities [38, 39]. Therefore, most PLWH in Ghana are afraid of disclosing of their HIV-positive status to their partners due to these negative perceptions and experiences [38, 39], thereby averting the negative repercussions of disclosure. From a practice standpoint, this finding may suggest that, while clinicians often advocate for status disclosures, they may need to consider the social implications of disclosure and plan for subsequent psychological outcomes associated with the disclosure of HIV status to the partner. However, this finding contrast the result of a study in Ethiopia which found that non-disclosure was associated with increased odds of depression [35].

We also found a positive association between stigma and depression. In Ghana, PLWH continue to experience various forms of stigma (Mumin et al., 2018), which ostensibly is associated with depression and decreased self-worth. This findings corroborates the results of previous studies in Ethiopia [35, 37, 40] and South Africa [41]. It is important to ensure that interventions address both stigma and depression among PLWH in Ghana.

Surprisingly, we found a positive association between social support and depressive symptoms with high social support associated with high depression. While the reason for this finding is unknown, it is possible that depressed PLWH received more social support. However, we are unable to explain the direction of the association. It is possible that greater social support may be associated with greater need or reliance upon others, which may decrease one’s self-worth. This findings contrast the results of a previous study in Ghana which found a negative association between social support and depression [34]. The findings also contrast the results of a previous study in Ethiopia which found that poor social support was positively associated depression [42]. We recommend that further studies using longitudinal approach to explore the relationship between depression and social support using longitudinal design to help understand the direction of the association.

There was a positive association between age and depression among PLWH with increasing age associated with high degree of depressive symptoms. This appears to contradict the assertion that older people are more able to adjust to the acute psychological stressors associated HIV infection [43], although we do not have data on the chronicity of HIV infection in our sample. In contrast, other studies have reported that younger PLWH experience higher depression than the older counterparts [35, 44].

The limitation of our study includes the use of a non-random sample and small sample size which limits the generalizability of our findings. In addition, the study used a cross sectional design, therefore no causality may be inferred from the observed association. Despite these limitations, our study has provided preliminary data to help understand the associated factors of depression among PLWH. Another limitation was the challenge with the large amount of missing data for some variables, which has a potential to bias our results if missing not at random (MNAR). We recommend that further studies use larger randomly selected participants sample to improve internal and external validity.

## Conclusion

Our study found 20.4% prevalence of depression among PLWH in in the Volta region of Ghana. It was revealed in our study that disclosure status, gender, level of education, age, social support, and stigma were significant independent predictors of depressive symptoms among PLWH in Ghana. The findings of our study suggest further research using a larger randomly selected participant with a longitudinal study design to understand causes of depression among PLWH in Ghana.

## Supporting information

S1 Checklist PLoS inclusivity in global research checklist. (DOCX)
